# Efficacy and safety of denosumab and teriparatide versus oral bisphosphonates to treat postmenopausal osteoporosis: a systematic review and meta-analysis

**DOI:** 10.3389/fendo.2024.1431676

**Published:** 2024-09-02

**Authors:** Jia Yang, Xiaobo Guo, Zhongning Cui, Huikang Guo, Jia-Nan Dong

**Affiliations:** ^1^ Department of Orthopedics, Jincheng General Hospital, Jincheng, China; ^2^ Department of Gynecology, Gaoping People's Hospital, Jincheng, China

**Keywords:** postmenopausal osteoporosis, denosumab, teriparatide, oral bisphosphonates, efficacy & safety

## Abstract

**Study Design:**

A systematic review and Meta-analysis

**Objective:**

To compare the efficacy and safety of denosumab and teriparatide versus oral bisphosphonates to treat postmenopausal osteoporosis.

**Summary of Background Data:**

While bisphosphonates have historically been the cornerstone of pharmacological management for bone protection in patients, emerging evidence suggests that teriparatide and denosumab warrant further investigation as potential first-line treatments. The optimal choice among denosumab, teriparatide, and oral bisphosphonates for the treatment of postmenopausal osteoporosis remains a subject of ongoing debate and controversy within the scientific community.

**Methods:**

This systematic review adhered meticulously to the rigorous standards outlined by the PRISMA (Preferred Reporting Items for Systematic Reviews and Meta-Analysis) guidelines as well as the Cochrane Collaboration recommendations. Additionally, it employed the AMSTAR (Assessing the methodological quality of systematic reviews) criteria to ensure methodological robustness and enhance the credibility of the findings. A systematic electronic search was conducted across Web of Science, PubMed, and the Cochrane Library databases from their inception dates up to February 2024.

**Results:**

In this meta-analysis of studies, our findings suggest that compared to bisphosphonates, both teriparatide and denosumab demonstrated notable increases in percentage changes in lumbar spine bone mineral density (BMD) among postmenopausal osteoporosis patients. Furthermore, denosumab exhibited superiority over teriparatide and oral bisphosphonates in enhancing percentage changes in both femoral neck and total hip BMD, indicating its potential as a more efficacious option. Regarding safety outcomes, no significant differences were observed in the incidence of serious adverse events among patients treated with teriparatide, denosumab, and bisphosphonates. However, teriparatide showed superiority over oral bisphosphonates in terms of a lower risk of general adverse events, suggesting a favorable safety profile.

**Conclusion:**

In conclusion, our study suggests that teriparatide and denosumab demonstrate comparable or potentially superior efficacy and safety profiles compared to oral bisphosphonates for the treatment of postmenopausal osteoporosis.

**Systematic Review Registration:**

PROSPERO, identifier CRD42024508382.

## 1 Introduction

Osteoporosis, characterized as a systemic bone disease, primarily entails the depletion of bone mass and degradation of bone tissue microstructure. This process significantly heightens bone fragility, thereby elevating the risk of fractures (1, 2). In women, the decline in estrogen levels post-menopause, a hormone known for its bone-protective effects, contributes to the onset of osteoporosis, substantially augmenting fracture risk (3–5). It has been reported that approximately 30% of women in the United States are predisposed to developing osteoporosis (6). Therefore, postmenopausal osteoporosis imposes a significant burden on both individual patients and society as a whole.

Bisphosphonates is the most commonly prescribed and available drugs worldwide. Nevertheless, the extended usage of these drugs and their potential adverse effects have sparked ongoing debate within the scientific community (7–9). Denosumab, a fully human monoclonal antibody targeting receptor activator of nuclear factor kappa-B ligand (RANKL), demonstrates efficacy in mitigating bone resorption while concurrently enhancing bone mineral density (BMD) (10, 11). Denosumab received its initial approval from the United States Food and Drug Administration (FDA) in 2010 for the treatment of postmenopausal osteoporosis in high-risk individuals prone to fractures (12). Teriparatide is a synthetic form of human parathyroid hormone (1–34), generated through recombinant technology (13, 14). This implies that the medication can trigger bone remodeling by enhancing osteoblast activity, resulting in a significant elevation in the bone formation marker P1NP. Ultimately, this process culminates in heightened bone density, effectively achieving the therapeutic goal of treating osteoporosis (15).

While bisphosphonates have historically served as the cornerstone of bone protective therapy, teriparatide and denosumab are gaining recognition as promising first-line treatments, warranting further investigation. Consequently, there remains a contentious debate regarding the optimal choice between denosumab, teriparatide, or bisphosphonates for postmenopausal osteoporosis management. The objective of this meta-analysis is to comprehensively evaluate the efficacy and safety profiles of denosumab and teriparatide versus oral bisphosphonates in the treatment of postmenopausal osteoporosis.

## 2 Materials and methods

### 2.1 Search strategy and selection criteria

This systematic review adhered meticulously to the rigorous standards outlined by the PRISMA (Preferred Reporting Items for Systematic Reviews and Meta-Analysis) guidelines as well as the Cochrane Collaboration recommendations. Additionally, it employed the AMSTAR (Assessing the methodological quality of systematic reviews) criteria to ensure methodological robustness and enhance the credibility of the findings (16–18). The methodologies employed in this review were pre-registered with PROSPERO under registration number CRD42024508382. A systematic electronic search was conducted across Web of Science, PubMed, and the Cochrane Library databases from their inception dates up to February 2024. The comprehensive search strategy is detailed in (**Supplementary Data**). Additionally, manual searches of reference lists from pertinent reviews and included studies were performed to ensure thoroughness in data collection.

### 2.2 Selection criteria and study design

The inclusion criteria for this review encompassed randomized controlled trials with a minimum duration of 12 months involving postmenopausal osteoporosis patients. Studies were eligible if they compared either denosumab or teriparatide with a single oral bisphosphonate drug. (Due to the current lack of randomized controlled trials comparing denosumab and teriparatide with intravenous bisphosphonates, intravenous bisphosphonates are not included in the scope.) Additionally, the selected literature needed to present at least one relevant outcome of interest.

Percentage changes in lumbar spine, total hip, and femoral neck bone mineral density (BMD) served as efficacy criteria. Firstly, lumbar spine bone mineral density (BMD) refers to the measurement of mineral content within the bones of the lumbar spine, typically assessed through densitometry techniques such as dual-energy X-ray absorptiometry (DXA). It provides information about the density and strength of the vertebrae in the lower back region (19). Then, total hip bone mineral density (BMD) refers to the measurement of mineral content within the bones of the entire hip joint, including the proximal femur and surrounding structures. This measurement is indicative of bone strength and density in the hip region (20). Finally, femoral neck bone mineral density (BMD) refers to the measurement of mineral content specifically within the narrow portion of the thigh bone (femur) known as the femoral neck. This measurement is particularly important as the femoral neck is a common site for hip fractures, and assessing its bone mineral density helps evaluate fracture risk and overall bone health (21).

Assessment of serious adverse events and general adverse events was utilized to evaluate safety outcomes. Serious adverse events denote notable and potentially life-threatening incidents that individuals may encounter during medical interventions or involvement in clinical trials. These events have the capacity to extend hospitalization periods, cause persistent or substantial disability, or in extreme cases, result in mortality (22). General adverse events, on the other hand, are negative health outcomes that are less severe compared to serious adverse events. They encompass a broad range of symptoms, from mild discomfort to moderate issues. General adverse events refer to undesirable outcomes or effects that arise during or after medical treatment, irrespective of the specific treatment or condition. These events may include a variety of issues, such as nausea, headaches, allergic reactions, or other complications, which are not directly related to the primary therapeutic goal of the treatment. For example, articles have described adverse events in the upper gastrointestinal (GI) tract associated with bisphosphonate use, such as nausea, vomiting, epigastric pain, and dyspepsia, reported shortly after the introduction of oral formulations of these drugs for osteoporosis treatment.

### 2.3 Data extraction and quality assessment

Two authors (JY and XBG) conducted data extraction, documenting details such as the first author’s name, year of publication, sample size, mean age of patients, dosage and interval of comparison, follow-up duration, and study design. Any discrepancies between the two authors were resolved through consensus with a third investigator (JLD). Data were compiled into an electronic database for analysis. The Cochrane Collaboration risk assessment tool for randomized controlled trials (RCTs) was employed to evaluate the quality of the included literature (23, 24). Studies were evaluated for risk of bias based on several criteria including random sequence generation, allocation concealment, blinding of participants and personnel, blinding of outcome assessment, incomplete outcome data, selective reporting, and other potential biases. Ratings of low risk, high risk, or unclear risk were assigned accordingly. Any discrepancies between ratings were resolved through discussion between two independent authors, with a third investigator reviewing and finalizing decisions.

### 2.4 Data synthesis and statistical analysis

Statistical analyses were conducted using Review Manager software (version 5.3) (25). Data were summarized utilizing odds ratios (OR) for categorical variables and mean differences (MDs) for continuous data (26). Significance was set at P < 0.05. Heterogeneity among studies was evaluated using the I^2^ test, with interpretation categorized as absent (0%–25%), low (25.1%–50%), moderate (50.1%–75%), or high (75.1%–100%) (27). Funnel plots were utilized to assess publication bias, while forest plots were employed to visually depict individual study results and the corresponding effect sizes of combined estimates.

## 3 Results

### 3.1 Systematic review and qualitative assessment

**Figure 1:**
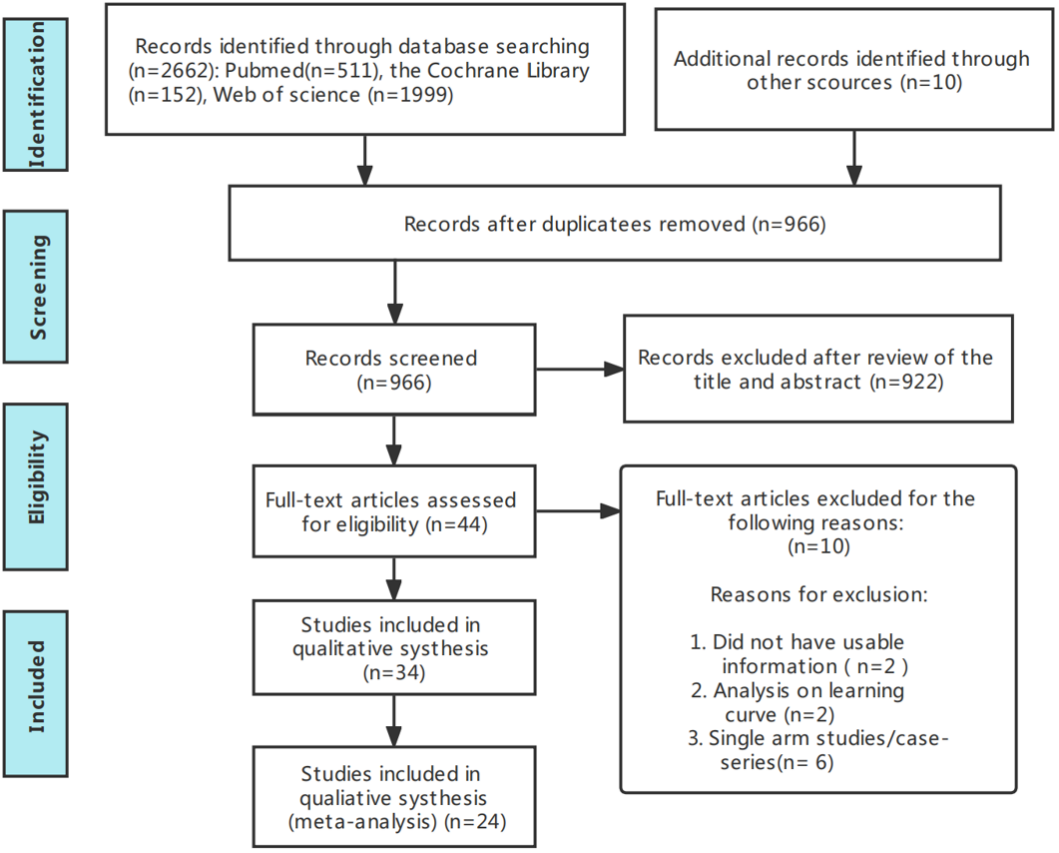
Flow chart of the selection process for relative studies in meta-analysis.

**Figure 2:**
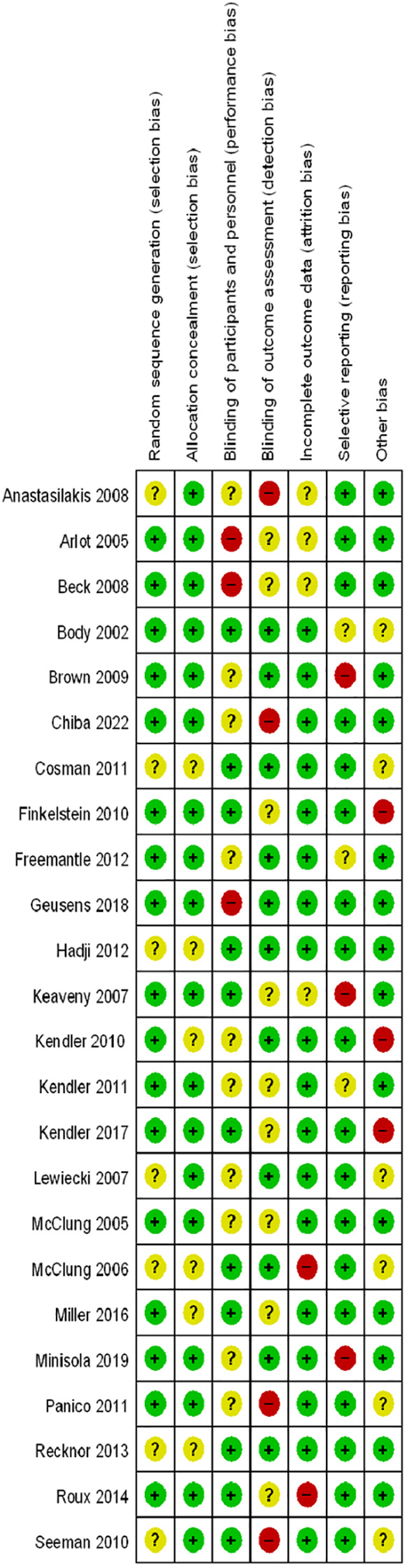
Risk of bias summary for RCTs: Reviewers’ judgments about each risk of bias item per included study.

**Figure 1** presents the flowchart according to the PRISMA statement, illustrating the study selection process alongside the primary exclusion criteria. Ultimately, our analysis incorporated 24 randomized controlled trials (28–51). Further insights into the risk of bias are encapsulated in **Figure 2**.

### 3.2 Trials characteristics

**Tables 1**, **2** encapsulate the primary characteristics of the trials incorporated in our analysis. These trials span the publication period from 2002 to 2024. Within the teriparatide and bisphosphonates cohort, 5 out of 13 trials conducted comparative assessments between teriparatide and risedronate, while seven trials scrutinized the efficacy of teriparatide against alendronate, and one trial investigated its comparison with zoledronic acid. In the denosumab and oral bisphosphonates subgroup, 1 out of 11 trials compared denosumab with risedronate, eight evaluated denosumab against alendronate, one assessed denosumab versus zoledronic acid, and one investigated denosumab versus ibandronate.

**Table 1:** The Main Features of the Articles Between the Teriparatide and Bisphosphonates.

	Sample (*n*)		Mean age (year)		Dose and interval		Follow-up	Study
Author	Intervention	Comparison	Intervention		Intervention	Comparison		
Body 2002 (51)	73	73	66	65	Teriparatide Sc 40 μg daily	alendronate oral 70 mg once weekly	12M	RCTs
Arlot 2005 (50)	21	21	60.9	65.5	Teriparatide Sc 20 μg daily	alendronate oral 70 mg once weekly	18M	RCTs
McClung 2005 (49)	102	101	65.3	66.6	Teriparatide Sc 20 μg daily	alendronate oral 70 mg once weekly	18M	RCTs
Keaveny 2007 (47)	28	25	64.5	62.5	Teriparatide Sc 20 μg daily	alendronate oral 70 mg once weekly	18M	RCTs
Anastasilakis 2008 (45)	22	22	65.4	64.7	Teriparatide Sc 20 μg daily	risedronate oral 35 mg once weekly	12M	RCTs
Finkelstein 2010 (42)	20	29	65	64	Teriparatide Sc 40 μg daily	alendronate oral 70 mg once weekly	30M	RCTs
Cosman 2011 (39)	138	137	63.8	66.1	Teriparatide Sc 20 μg daily	zoledronic 5 mg	12M	RCTs
Panico 2011 (37)	42	39	65	60	Teriparatide Sc 20 μg daily	alendronate oral 70 mg once weekly	18M	RCTs
Hadji 2012 (35)	360	350	70.5	71.6	Teriparatide Sc 20 μg daily	risedronate oral 35 mg once weekly	12M	RCTs
Kendler 2017 (30)	680	680	72.6	71.6	Teriparatide Sc 20 μg daily	risedronate oral 35 mg once weekly	24M	RCTs
Geusens 2018 (31)	680	680	NS	NS	Teriparatide Sc 20 μg daily	risedronate oral 35 mg once weekly	24M	RCTs
Minisola 2019 (29)	114	119	72.6	71.6	Teriparatide Sc 20 μg daily	risedronate oral 35 mg once weekly	24M	RCTs
Chiba 2022 (28)	33	38	71.7	71.9	Teriparatide Sc 20 μg daily	alendronate/risedronate oral 35/17.5 mg once weekly	18M	RCTs

**Table 2:** The Main Features of the Articles Between the Denosumab and Bisphosphonates.

	Sample (n)		Mean age (year)		Dose and interval		Follow-up	Study
Author	Intervention	Comparison	Intervention		Intervention	Comparison		
McClung 2006 (48)	47	47	63.1	62.8	denosumab Sc 60 mg Q6M	alendronate oral 70 mg once weekly	12M	RCTs
Lewiecki 2007 (46)	319	47	62.3	62.8	denosumab Sc 60 mg Q6M	alendronate oral 70 mg once weekly	24M	RCTs
Beck 2008 (44)	39	38	63	63	denosumab Sc 60 mg Q6M	alendronate oral 70 mg once weekly	24M	RCTs
Brown 2009 (43)	594	595	64.1	64.6	denosumab Sc 60 mg Q6M	alendronate oral 70 mg once weekly	12M	RCTs
Kendler 2010 (41)	253	251	66.9	68.2	denosumab Sc 60 mg Q6M	alendronate oral 70 mg once weekly	12M	RCTs
Seeman 2010 (40)	83	82	60.3	60.7	denosumab Sc 60 mg Q6M	alendronate oral 70 mg once weekly	12M	RCTs
Kendler 2011 (38)	253	251	66.9	68.2	denosumab Sc 60 mg Q6M	alendronate oral 70 mg once weekly	12M	RCTs
Freemantle 2012 (36)	126	124	65.1	65.3	denosumab Sc 60 mg Q6M	alendronate oral 70 mg once weekly	12M	RCTs
Recknor 2013 (34)	417	416	67.2	66.2	denosumab Sc 60 mg Q6M	ibandronate oral 150 mg once month	12M	RCTs
Roux 2014 (33)	422	402	67.8	67.7	denosumab Sc 60 mg Q6M	risedronate oral 150 mg once month	12M	RCTs
Miller 2016 (32)	321	322	68.5	69.5	denosumab Sc 60 mg Q6M	Zoledronic 5 mg	12M	RCTs

### 3.3 Percentage changes in the lumbar spine BMD

**Figure 3:**
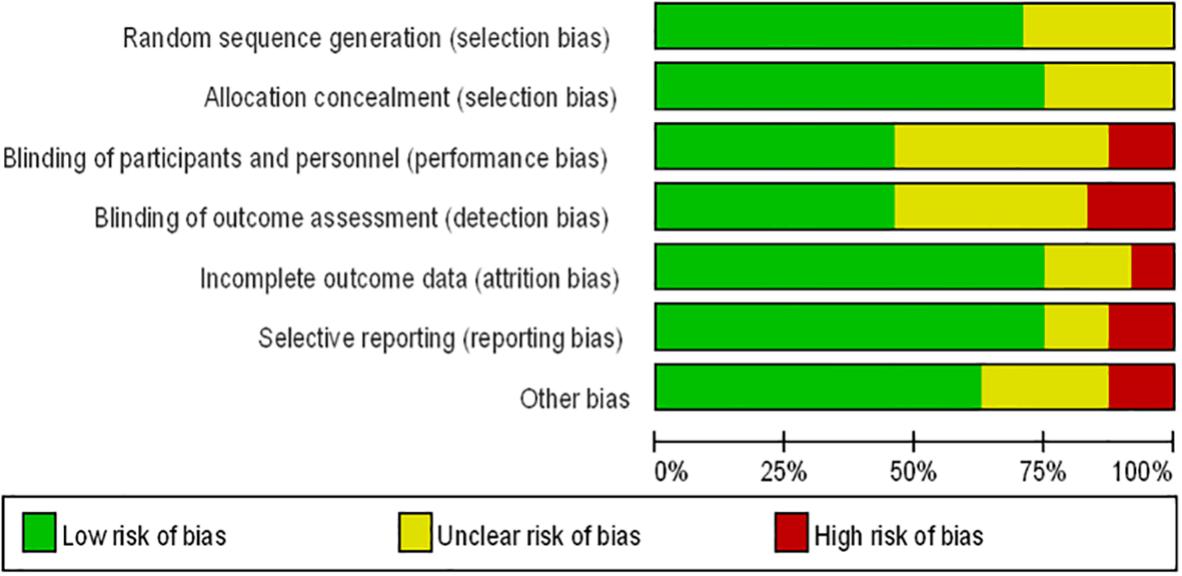
The forest plots in percentage changes in the lumbar spine BMD.

**Figure 3** presents forest plots depicting the percentage changes in lumbar spine bone mineral density (BMD). The data illustrate that compared to bisphosphonates, both teriparatide and denosumab led to further increases in percentage changes in lumbar spine BMD among postmenopausal osteoporosis patients [teriparatide arm: RR=5.16, 95%CI:5.09–5.24, P < 0.00001; denosumab arm: RR=1.21, 95%CI: 0.3–2.11, p=0.009]. Notably, there was no significant statistical heterogeneity observed between the teriparatide and oral bisphosphonates results (I^2^ = 0%). However, the denosumab and oral bisphosphonates group exhibited substantial heterogeneity (I^2^ = 97%).

### 3.4 Percentage changes in the femoral neck BMD

**Figure 4:**
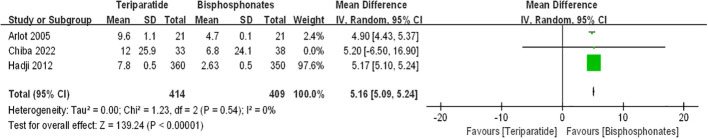
The forest plots in percentage changes in the femoral neck BMD.

**Figure 4** displays forest plots illustrating the percentage changes in femoral neck bone mineral density (BMD). The data indicate that compared to bisphosphonates, denosumab led to a further increase in percentage changes in femoral neck BMD among postmenopausal osteoporosis patients [denosumab arm: RR=1.03, 95%CI: 0.69–1.37, P < 0.00001]. However, the difference in percentage changes in femoral neck BMD between the teriparatide and oral bisphosphonates groups did not reach statistical significance [teriparatide arm: RR=0.74, 95%CI:-0.46–1.94, p=0.23]. Notably, there was substantial statistical heterogeneity observed between the two groups of results (I^2^ = 76%, I^2^ = 88%). Thus, we advocate for cautious interpretation of these findings, and we emphasize the necessity for additional randomized controlled trials to validate these results.

### 3.5 Percentage changes in the total hip BMD

**Figure 5:**
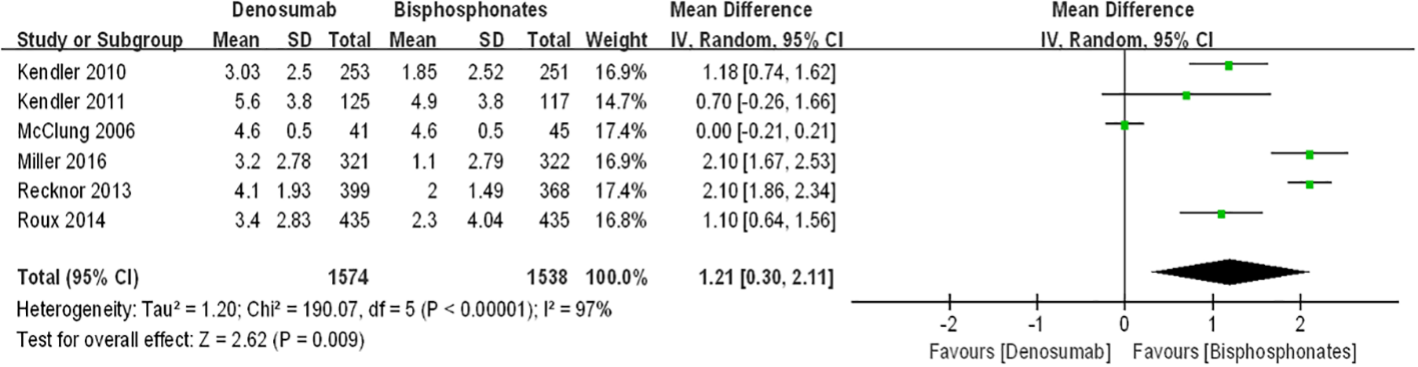
The forest plots in percentage changes in the total hip BMD.

**Figure 5** depicts forest plots presenting the percentage changes in total hip bone mineral density (BMD). The data reveal that compared to bisphosphonates, denosumab induced a further increase in percentage changes in total hip BMD among postmenopausal osteoporosis patients [denosumab arm: RR=0.83, 95%CI:0.50–1.17, P < 0.00001]. Conversely, the difference in percentage changes in total hip BMD between the teriparatide and oral bisphosphonates groups did not reach statistical significance [teriparatide arm: RR=0.83, 95%CI:-0.28–1.94, p=0.15]. Noteworthy, there was substantial statistical heterogeneity observed between the denosumab and oral bisphosphonates groups (I^2^ = 81%), while the teriparatide and oral bisphosphonates group exhibited moderate heterogeneity (I^2^ = 63%).

### 3.6 General adverse events

**Figure 6:**
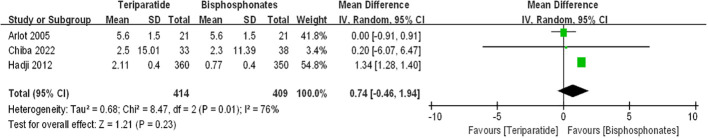
The forest plots in general adverse events.

**Figure 6** presents forest plots illustrating the incidence of general adverse events. The data indicate that compared to teriparatide, the risk of general adverse events was statistically higher with oral bisphosphonates [teriparatide arm: RR=0.69, 95%CI: 0.49–0.97, p=0.04]. Conversely, there was no significant difference in the incidence of general adverse events between the denosumab and oral bisphosphonates groups [denosumab arm: RR=1.06, 95%CI:0.93–1.21, p=0.37]. Notably, there was no statistical heterogeneity observed between the denosumab and oral bisphosphonates groups (I^2^ = 0), while the teriparatide and oral bisphosphonates group displayed moderate heterogeneity (I^2^ = 55%). These findings underscore the importance of cautious interpretation and further investigation into the safety profiles of these treatments.

### 3.7 Serious adverse events

**Figure 7:**
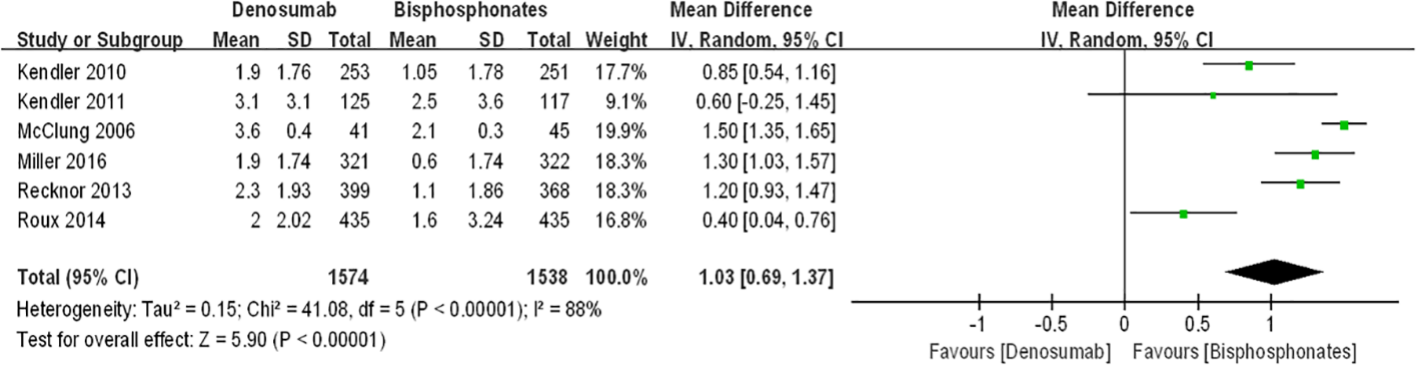
The forest plots in serious adverse events.

**Figure 7** illustrates forest plots depicting the incidence of serious adverse events. The data reveal that there were no significant differences in the incidence of serious adverse events among patients treated with teriparatide, denosumab, and oral bisphosphonates [teriparatide arm: RR=1.01, 95%CI:0.65–1.57, p=0.95; denosumab arm: RR=1.04, 95%CI:0.79–1.37, p=0.80]. Noteworthy, there was no statistical heterogeneity observed between the denosumab and oral bisphosphonates groups (I^2^ = 18%), while the teriparatide and oral bisphosphonates group exhibited moderate heterogeneity (I^2^ = 70%). These findings suggest a comparable safety profile among these treatments in terms of serious adverse events, albeit with some variability that warrants further investigation.

## 4 Discussion

In this systematic review and meta-analysis, we offer a comprehensive overview of the efficacy and safety profiles of denosumab and teriparatide versus oral bisphosphonates in the treatment of postmenopausal osteoporosis. Our analysis provides valuable insights into the comparative effectiveness and safety considerations of these therapeutic interventions.

In this meta-analysis of studies, our findings suggest that compared to oral bisphosphonates, both teriparatide and denosumab demonstrated notable increases in percentage changes in lumbar spine bone mineral density (BMD) among postmenopausal osteoporosis patients. Furthermore, denosumab exhibited superiority over teriparatide and oral bisphosphonates in enhancing percentage changes in both femoral neck and total hip BMD, indicating its potential as a more efficacious option. Regarding safety outcomes, no significant differences were observed in the incidence of serious adverse events among patients treated with teriparatide, denosumab, and bisphosphonates. However, teriparatide showed superiority over oral bisphosphonates in terms of a lower risk of general adverse events, suggesting a favorable safety profile. This appears to align with the descriptions provided by Chandran et al. And Yuan et al. Chandran et al. described in their article that denosumab can serve as both a first-line agent and an alternative to bisphosphonates for treating postmenopausal osteoporosis (52). Yuan et al. suggested that compared to bisphosphonates, teriparatide can reduce the risk of vertebral fractures and increase changes in lumbar spine and femoral neck BMD (6).

In summary, our analysis suggests that both teriparatide and denosumab exhibit similar or even superior efficacy and safety profiles compared to oral bisphosphonates for the treatment of postmenopausal osteoporosis. One interesting point to consider is that while this study found denosumab to be more effective than oral bisphosphonates overall in increasing bone mineral density (BMD), particularly in the femoral neck and total hip, its effect on lumbar spine BMD appeared to be similar to that of teriparatide. This suggests that different treatment medications may have varying effects on different skeletal regions, highlighting the importance of considering individual patient characteristics and the location of BMD changes when making treatment decisions. Furthermore, despite denosumab and teriparatide showing similar effects in increasing BMD, denosumab seems to exhibit higher heterogeneity in some aspects. This may indicate that the efficacy of denosumab across different studies could be influenced by other factors such as baseline characteristics of patients or duration of treatment. The presence of this heterogeneity may necessitate further research to determine the true effect of denosumab and its optimal application in clinical practice.

These findings provide valuable insights for clinicians in selecting optimal therapeutic options for their patients. Our meta-analysis had some advantages. To the best of our knowledge, we are the first study to combine denosumab, teriparatide and bisphosphonate in a direct comparison study in postmenopausal osteoporosis, which means that unlike the network meta-analysis, the results of the direct comparison will be more reliable. At the same time, a large number of studies (n = 24) including data from more 9000 patients were included, and all these studies were RCTs, which makes the results more credible.

This paper acknowledges several limitations. Firstly, the wide time span of included literature, ranging from 2002 to 2024, may introduce variability in study methodologies and patient characteristics, potentially impacting the quality of the meta-analysis. Secondly, the limited availability of literature specifically addressing serious adverse events and percentage changes in total hip BMD within the teriparatide and oral bisphosphonates group could restrict the robustness of the final analysis results. Finally, This article primarily discusses oral bisphosphonates. This is partly due to insufficient RCTs for intravenous bisphosphonates. On the other hand, oral and intravenous bisphosphonate administrations differ fundamentally. Intravenous treatment is generally preferred over oral bisphosphonates due to its perceived ease, efficacy, reduced burden, lower opportunity costs. However, Abhishek Sharma et al. suggest that intravenous bisphosphonates might increase inflammatory cytokine release more than oral bisphosphonates, potentially increasing the risk of new-onset atrial fibrillation (53). Furthermore, upper gastrointestinal discomfort from oral bisphosphonates and acute reactions from intravenous formulations are significant considerations (54). These factors underscore the need to evaluate both oral and intravenous administration of bisphosphonates. Moving forward, it is imperative to conduct additional studies aimed at generating high-quality randomized controlled trials (RCTs) on these topics. This will facilitate the acquisition of more reliable analysis results, thereby enhancing our understanding of the efficacy and safety profiles of these therapeutic interventions for postmenopausal osteoporosis.

## 5 Conclusion

In conclusion, our study suggests that teriparatide and denosumab demonstrate comparable or potentially superior efficacy and safety profiles compared to oral bisphosphonates for the treatment of postmenopausal osteoporosis. We believe that they hold promise as potential first-line treatments for this condition. However, given the absence of clear standards and the inherent variability in individual physiological conditions, further high-quality research is warranted to comprehensively explore the efficacy and safety of denosumab, teriparatide, and oral bisphosphonates in the treatment of postmenopausal osteoporosis, ensuring safety under varied clinical circumstances.

## Data Availability

The original contributions presented in the study are included in the article/**Supplementary Material**. Further inquiries can be directed to the corresponding author.
