# Supplementary material for: Efficacy and safety of denosumab and teriparatide versus oral bisphosphonates to treat postmenopausal osteoporosis: a systematic review and meta-analysis
Source: Front Endocrinol (Lausanne). 2024 Sep 2;15:1431676. doi: 10.3389/fendo.2024.1431676 (PMC11402677; doi:10.3389/fendo.2024.1431676)
Supplement: Supplementary file 1 [file DataSheet1.docx]

**Appendix.**

**Supplementary data**

**Search Strategy;**

**PUBMED （33）**

(((((((((((((((((Osteoporosis, Postmenopausal[MeSH Terms]) OR (Perimenopausal Bone Loss[Title/Abstract])) OR (Bone Loss, Postmenopausal[Title/Abstract])) OR (Bone Losses, Postmenopausal[Title/Abstract])) OR (Postmenopausal Bone Losses[Title/Abstract])) OR (Osteoporosis, Post-Menopausal[Title/Abstract])) OR (Osteoporoses, Post-Menopausal[Title/Abstract])) OR (Osteoporosis, Post Menopausal[Title/Abstract])) OR (Post-Menopausal Osteoporoses[Title/Abstract])) OR (Post-Menopausal Osteoporosis[Title/Abstract])) OR (Postmenopausal Osteoporosis[Title/Abstract])) OR (Osteoporoses, Postmenopausal[Title/Abstract])) OR (Postmenopausal Osteoporoses[Title/Abstract])) OR (Bone Losses, Perimenopausal[Title/Abstract])) OR (Perimenopausal Bone Losses[Title/Abstract])) OR (Postmenopausal Bone Loss[Title/Abstract])) AND ((((Teriparatide[MeSH Terms]) OR (denosumab[MeSH Terms])) OR (Teriparatide Acetate[Title/Abstract])) OR (Forteo[Title/Abstract]))) AND (((((Diphosphonate[MeSH Terms]) OR (Bisphosphonate[Title/Abstract])) OR (alendronate[Title/Abstract])) OR (ibandronate[Title/Abstract])) OR (zoledronic[Title/Abstract]))

**Cochrane Central Register of Controlled Trials**

#1 MeSH descriptor: [Osteoporosis, Postmenopausal] explode all trees

#2 (Perimenopausal Bone Loss):ti,ab,kw OR (Bone Loss, Postmenopausal):ti,ab,kw OR (Bone Losses, Postmenopausal):ti,ab,kw OR (Postmenopausal Bone Losses):ti,ab,kw OR (Osteoporosis, Post-Menopausal):ti,ab,kw

#3 (Osteoporoses, Post-Menopausal):ti,ab,kw OR (Osteoporosis, Post Menopausal):ti,ab,kw OR (Post-Menopausal Osteoporoses):ti,ab,kw OR (Post-Menopausal Osteoporosis):ti,ab,kw OR (Postmenopausal Osteoporosis):ti,ab,kw

#4 (Osteoporoses, Postmenopausal):ti,ab,kw OR (Postmenopausal Osteoporoses):ti,ab,kw OR (Bone Losses, Perimenopausal):ti,ab,kw OR (Perimenopausal Bone Losses):ti,ab,kw OR (Postmenopausal Bone Loss):ti,ab,kw

#5 #1 AND #2 OR #3 OR #4

#6 MeSH descriptor: [Teriparatide] explode all trees

#7 MeSH descriptor: [Denosumab] explode all trees

#8 MeSH descriptor: [Diphosphonates] explode all trees

#9 (Bisphosphonate):ti,ab,kw OR (alendronate):ti,ab,kw OR (ibandronate):ti,ab,kw OR (zoledronic):ti,ab,kw

#10 #6 OR #7

#11 #8 OR #9

#12 #5 AND #10 AND #11

**Web of science**

1: (((TS=(Teriparatide)) OR TS=(denosumab)) OR TS=(Teriparatide Acetate)) OR TS=(Forteo)

2: ((((TS=(Diphosphonate)) OR TS=(Bisphosphonate)) OR TS=(alendronate)) OR TS=(ibandronate)) OR TS=(zoledronic)

3: (((((((((((((((TS=(Osteoporosis, Postmenopausal)) OR TS=(Perimenopausal Bone Loss)) OR TS=(Bone Loss, Postmenopausal)) OR TS=(Bone Losses, Postmenopausal)) OR TS=(Postmenopausal Bone Losses)) OR TS=(Osteoporosis, Post-Menopausal)) OR TS=(Osteoporoses, Post-Menopausal)) OR TS=(Osteoporosis, Post Menopausal)) OR TS=(Post-Menopausal Osteoporoses)) OR TS=(Post-Menopausal Osteoporosis)) OR TS=(Postmenopausal Osteoporosis)) OR TS=(Osteoporoses, Postmenopausal)) OR TS=(Postmenopausal Osteoporoses)) OR TS=(Bone Losses, Perimenopausal)) OR TS=(Perimenopausal Bone Losses)) OR TS=(Postmenopausal Bone Loss)

4: #3 AND #2 AND #1

**Supplementary Figures;**

**Supplementary Figure 1：**Funnel plot of publication bias in the the percentage changes in the lumbar spine BMD.

**Supplementary Figure 2：**Funnel plot of publication bias in the the percentage changes in the femoral neck BMD.

**Supplementary Figure 3：**Funnel plot of publication bias in the the percentage changes in the total hip BMD.

**Supplementary Figure 4：**Funnel plot of publication bias in the the percentage changes in the general adverse events.

**Supplementary Figure 5：**Funnel plot of publication bias in the the percentage changes in the serious adverse events.
